# Suppressor of Cytokine Signaling-2 (SOCS2) Regulates the Microglial Response and Improves Functional Outcome after Traumatic Brain Injury in Mice

**DOI:** 10.1371/journal.pone.0153418

**Published:** 2016-04-12

**Authors:** Harleen S. Basrai, Kimberly J. Christie, Alisa Turbic, Nicole Bye, Ann M. Turnley

**Affiliations:** Department of Anatomy and Neuroscience, The University of Melbourne, Parkville, Victoria, Australia; Universidade de São Paulo, BRAZIL

## Abstract

Traumatic brain injury (TBI) is frequently characterized by neuronal, axonal and myelin loss, reactive gliosis and neuroinflammation, often associated with functional deficits. Endogenous repair mechanisms include production of new neurons from precursor cells, but usually the new neurons fail to integrate and survive more than a few weeks. This is in part mediated by the toxic and inflammatory environment present in the injured brain which activates precursor cells to proliferate and differentiate but limits survival of the newborn progeny. Therefore, an understanding of mechanisms that regulate production and survival of newborn neurons and the neuroinflammatory response after brain injury may lead to therapeutic options to improve outcomes. Suppressor of Cytokine Signaling 2 (SOCS2) promotes hippocampal neurogenesis and survival of newborn neurons in the adult brain and regulates anti-inflammatory responses in the periphery, suggesting it may be a useful candidate to improve outcomes of TBI. In this study the functional and cellular responses of SOCS2 over-expressing transgenic (SOCS2Tg) mice were compared to wildtype littermates following mild or moderately severe TBI. Unlike wildtype controls, SOCS2Tg mice showed functional improvement on a ladder test, with a smaller lesion volume at 7d post injury and increased numbers of proliferative CD11b^+^ microglia/macrophages at 35d post-injury in the mild injury paradigm. At 7d post-moderately severe injury there was an increase in the area covered by cells expressing an anti-inflammatory M2 phenotype marker (CD206^+^) but no difference in cells with a pro-inflammatory M1 phenotype marker (CD16/32^+^). No effect of SOCS2 overexpression was observed in production or survival of newborn neurons, even in the presence of the neuroprotective agent erythropoietin (EPO). Therefore, SOCS2 may improve outcome of TBI in mice by regulating aspects of the neuroinflammatory response, promoting a more anti-inflammatory environment, although this was not sufficient to enhance survival of newborn cortical neurons.

## Introduction

Traumatic brain injury (TBI) affects millions of people worldwide. In the United Sates alone up to 1.7 million people report to have sustained a TBI annually [1]. Despite its high prevalence and socioeconomic impact, strategies for treatment of TBI are lacking. Primary trauma and secondary neuroinflammatory events lead to neuronal loss and this is accompanied by a range of behavioral and cognitive deficits whose exact nature and extent varies depending on the location, mode and severity of injury [2, 3]. Replacement of lost neurons to recover function/s after TBI remains an important bottleneck in the development of effective therapeutic treatments. Strategies under investigation to overcome this bottleneck include exogenous neural precursor cell (NPC) transplantation and also stimulation of endogenous adult neurogenesis.

### Adult neurogenesis and brain injury

Endogenous adult neurogenesis occurs throughout life in the adult brain and is localized to the sub-ventricular zone (SVZ) of the lateral ventricles and the sub-granular zone (SGZ) of hippocampal dentate gyrus [4]. It is now well established that endogenous adult neurogenesis responds to TBI and evidence for this exists in rodents, swine, non-human primates and humans [5–8]. It is still controversial, however, if injury-induced NPC proliferation is associated with an increase in newborn neurons after TBI. Indeed in a rat model of fluid percussion injury which produced a cognitive functional deficit, the time point of spontaneous cognitive functional recovery coincided with an increased number of integrated newborn neurons in the GCL of the dentate gyrus [9]. However, a recent study in mice after a controlled cortical impact injury, found only enhanced NPC proliferation at the SGZ and no associated increase in integrated newborn neurons in the GCL [10]. TBI also enhances NPC proliferation at the SVZ of the lateral ventricles and SVZ derived NPCs/neuroblasts are re-directed from their normal migratory path towards the OB to instead migrate towards the cortical lesion site [6, 11, 12]. Similar to injury-induced adult hippocampal neurogenesis, evidence for these newly proliferated migrating NPCs surviving to become newborn neurons at the lesion site is mixed. Indeed only a handful of studies have identified limited numbers of newborn neurons in the perilesional cortex after TBI [12–14]. These findings suggest that endogenous adult neurogenesis has the potential to be reparative after TBI but needs to be aided with appropriate factors that can stimulate sufficient NPC proliferation, provide an ideal microenvironment for successful NPC differentiation and importantly enhance newborn neuron survival.

### SOCS2 and neurogenesis

In terms of enhancing newborn neuron survival after TBI, Suppressor of Cytokine Signaling 2 (SOCS2) may be one such factor of interest. SOCS2 is a member of the SOCS protein family and is best characterized as a negative regulator of growth hormone (GH) signaling [15]. SOCS2 also has roles dependent [16] and independent of GH in the CNS [17]. It is a downstream regulator of Epidermal Growth Factor (EGF) and Nerve Growth Factor (NGF) signaling, promoting neurite [18–20]. Further, brains of adult SOCS2 overexpressing transgenic (SOCS2Tg) mice had increased cortical neuron density coupled with increased dendrite arborisation [21]. They also show increased adult hippocampal neurogenesis, with enhanced survival of newborn adult hippocampal neurons both under basal physiological and voluntary running conditions, independent of GH signaling [22]. In addition to EGF and NGF signaling, SOCS2 has been shown to associate with the Brain Derived Neurotrophic Factor (BDNF) receptor TrkB (unpublished observations) and to modulate erythropoietin signaling, both of which have pro-neurogenic roles and may play a role in SOCS2 regulation of neurogenesis [23–26].

SOCS2 overexpressing animals show enhanced survival of newborn adult hippocampal neurons under basal physiological conditions [22], while SOCS2KO animals have reduced numbers of surviving newborn adult hippocampal neurons (manuscript submitted). Interestingly, in a rat model of transient forebrain ischemia, an upregulation of SOCS2 expression in the GCL of the dentate gyrus has been reported to play a role in ischemia induced endogenous hippocampal neurogenesis [27]. In terms of cortical neuron replacement following TBI, SVZ rather than SGZ derived NPCs are of greater importance as only they are able to migrate to ectopic injury locations [14]. While the role for SOCS2 in the regulation of adult SVZ-olfactory bulb neurogenesis has not yet been studied, SOCS2 overexpressing mice show greater cortical neuron density and dendritic arborization under basal physiological conditions [28].

### TBI and glial responses

TBI not only stimulates endogenous neurogenesis but also activates oligodendrogenesis, astrogliogenesis and macrophage/microglial proliferation at the site of injury [29–31]. Similar to adult neurogenesis, oligodendrogenesis can replace oligodendrocytes lost to the injury by activation of oligodendrocyte precursor cells (OPCs) that proliferate and mature into new myelinating oligodendrocytes. Astrogliogenesis and macrophage/microglial activation form an acute protective immune response to TBI that aims to contain the lesion and associated debris such that the surrounding healthy tissue is protected from secondary damage [32, 33]. However, especially in cases of moderate to severe TBI, this initial protective inflammatory response can develop into chronic inflammation that exacerbates the injury by causing neurotoxicity [34–36]. Therefore, understanding and modulating the glial and inflammatory cell response is equally as important as understanding and modulating endogenous adult neurogenesis after TBI. Interestingly, there is evidence for a peripheral role of SOCS2 in regulating lipoxin mediated anti-inflammatory responses [37]. Therefore, to determine whether SOCS2 may similarly regulate neuroinflammatory responses, the glial and inflammatory cell response was also analyzed in SOCS2Tg animals after TBI.

### Erythropoietin (EPO), neural injury and SOCS2

EPO was originally described for its peripheral role in erythropoiesis in the bone marrow [38] but EPO and the EPO receptor are also expressed in the brain [39]. While the role of endogenous brain derived EPO remains to be further elucidated, EPO signaling has been shown to be important for the developing brain in the regulation of cell apoptosis and protection against oxidative stress [40]. Importantly, systemic EPO administration can have neuroprotective effects in various mouse models of brain injury by protecting against hypoxic damage, increasing neuron survival and/or reducing inflammatory cell responses [41–45]. Also, in the uninjured brain, systemic EPO administration induces a transient increase in hippocampal NPC proliferation [46]. Further, a relationship between SOCS2 and EPO has been suggested in a study where EPO induced neuronal differentiation of SVZ NPCs was associated with SOCS2 up-regulation [24]. Therefore, the neuroprotective effects of EPO observed in the literature and its potential pro-neurogenic relationship with SOCS2 suggest it may interact with SOCS2 to modulate effects following TBI.

### Aims of the study

This study aimed to determine whether SOCS2 overexpression modulated the outcome of TBI in mice. Our primary hypothesis was that SOCS2 would promote TBI-induced neurogenesis from SVZ-derived cells. However, given that SOCS2 can also regulate peripheral inflammatory responses and interact with EPO signaling, neuroinflammatory processes and the effect of EPO administration were also examined.

## Materials and Methods

### Animals

Mice were obtained from breeding colonies maintained at University of Melbourne. Animals were housed in a specific pathogen free facility in individually ventilated cages with food and water ad libitum and kept on a constant 12h light/dark cycle. The use of experimental animals was approved by the Animal Experimentation Ethics Committee of the Florey Institute of Neuroscience and Mental Health, University of Melbourne, Australia (Approval #: 11–083). SOCS2 transgenic (Tg) mice constitutively overexpress SOCS2 driven by the human ubiquitin C promoter in all tissues [47] and are on the C57Bl/6 background. Male and female mice hemizygous for the SOCS2 transgenic allele were used, with littermate WT mice as controls.

### EdU labelling of proliferative cells

To label proliferating cells in the brain the thymidine analogue 5-ethynyl-2’-deoxyuridine (EdU; Invitrogen (Life technologies), Melbourne, Australia) was used. EdU incorporates into the DNA of dividing cells during the S-phase of mitosis [48]. All animals were administered EdU via intra-peritoneal injection using a 30G insulin syringe (BD, Sydney, Australia) at a dose of 50 mg/kg in saline. Animals were administered one daily dose of EdU for 7 days and tissues taken either at 8d to assess Neural Precursor Cell (NPC) proliferation and neuroblast generation or at 35 days to assess survival of newborn neurons.

### Erythropoietin administration

Recombinant human erythropoietin-alpha (EPO; ProSpecBio, Ness-Ziona, Israel) was administered via intra-peritoneal injection at a dose of 5000IU/kg in saline. EPO or a saline control was administered with EdU as above in a single injection.

### Controlled Cortical Impact TBI surgery

The controlled cortical impact (CCI) model of brain injury was used. Animals (8–16 weeks old) were anaesthetised with 5% isoflurane in an induction chamber. Anaesthesia was maintained at 1–2% isoflurane following placement of the animal onto a stereotaxic frame. Fur on the head was shaved, the area cleaned with 80% ethanol and eyes moistened with Lacri-Lube (Allergan, Sydney, Australia). Meloxicam (1mg/kg; Troy laboratories, Sydney, Australia), was administered subcutaneously in the back of the animals for analgesia. An incision was made on the midline of the head to expose the skull. A flat 1.8mm diameter Ketron® PEEK impactor tip (Alternative Engineering, Melbourne, Australia) attached to a computer controlled LinMot® linear motor (LinMot, Spreitenbach, Switzerland) and stereotaxic frame (David Kopf Instruments, California, USA), was placed over the target injury site at +2.5mm lateral and 0mm to bregma. These coordinates target the left sensory motor cortex, resulting in a motor deficit of the right forepaw following injury. The impactor tip was used as a guide to mark a 3 mm diameter circle over the injury site and a fine drill (KF Technology, Roma, Italy) used to thin the bone along the marking until the circular bone piece could be lifted off. The impactor tip was then lowered onto the exposed dura until it just touched the surface and the linear motor activated to induce the injury, with an impact velocity of 5m/s and an impact depth of 2mm. A mild or moderate severity injury was induced by using a tip dwell time of 100ms or 1000ms respectively. Following injury the exposed dura was covered with a 3.4mm diameter plastic disc (depth adjustment spacer discs included in the ALZET Brain Infusion Kit 3, Bioscientific, Sydney, Australia) and secured with Loctite 454 (superglue) gel. The skin was closed with 5–0 silk sutures and the animal moved into a Thermacage (Datesand Ltd, Manchester, UK) animal warming cabinet set at 30°C for at least 30min to aid recovery.

### Behavioural analyses pre and post-TBI–Horizontal Ladder

Motor function of moderately injured mice was analysed using the horizontal ladder test. The apparatus consisted of a 100cm long horizontal ladder made of evenly spaced (1cm apart) rounded metal ladder rungs. The ladder was placed within a clear Plexiglas holding frame 50cm in height such that the ladder sat 30cm above the ground. For the test, mice were placed on one end of the ladder and recorded on video as they walked across 50cm of the ladder to the other end. The home cage of the mouse was placed at the ladder end to encourage their movement across the ladder. Mice were run across the ladder three times or until two good runs were recorded from a maximum of 5 runs. A run was considered good if the mouse ran across at a constant speed without pauses. The best two runs were used to count the number of foot faults made. A foot fault was defined as when the right forepaw slipped through the ladder rungs. Counts from the best two 50cm runs were summed and a score of foot faults made over 100cm of the horizontal ladder obtained for each animal. The scores were normalised by subtracting post-TBI scores from pre-TBI scores which were averaged to obtain a score for the group.

### Brain tissue dissection and sectioning

#### Transcardial perfusion and brain dissection

At 7d or 35d post-injury mice were anaesthetised with 300μl of equal parts Lethabarb (Sodium Pentobarbitone; Virbac, Sydney, Australia) and saline and perfused through the left ventricle with 0.1M Phosphate Buffered Saline (20mL) to flush out the blood, followed by 4% paraformaldehyde (PFA; Sigma) in PBS (20mL) for fixing the tissue. The brain was dissected out and placed in 4% PFA at 4°C overnight followed by 30% sucrose (Chem-Supply Pty Ltd.) in PBS at 4°C for 48h.

Brains were hemisectioned with the aid of a mouse brain blocker (David Kopf Instruments, Sydney, NSW). The coronal cut was made at slot number 5 such that the front half of the tissue block contained lateral ventricles and the other half the hippocampus. Brains were placed cut side down into plastic Tissue-Tek® Cryomolds (Grale Scientific Pty Ltd., Australia), covered in in Tissue-Tek® optimum cutting temperature compound (O.C.T.; Grale Scientific Pty Ltd.), frozen in isopentane cooled over dry ice and stored at −80°C until use.

#### Brain tissue sectioning

Sectioning of brain tissue was done using a Leica cryostat. All free floating sections were collected in 24 well plates containing 500μl PBS. Free floating serial coronal sections, 30μm thick were collected through the injury site (bregma −1.2mm to 1.20mm). For each animal, 9 wells containing 6–8 sections spaced 300μm apart were collected. Every 10^th^ consecutive section was collected onto a Superfrost® Plus slide (Grale Scientific Pty Ltd.) for Hemotoxylin and Eosin staining. Following collection of all free floating sections, PBS was replaced with 500μl of anti-freeze (15% sucrose (Chem-Supply Pty Ltd.) and 30% Ethylene Glycol (Chem-Supply Pty Ltd.) in PBS) and sections stored at −20°C until staining.

### Immunohistochemistry and cell staining

#### Single marker immunohistochemistry

Floating serial sections were washed in PBS three times for at least 5min at RT. Sections were blocked with 5% normal goat serum (Invitrogen) or normal donkey serum (Sigma) and 0.02% Triton X-100 (Sigma) in PBS for at least 1h at RT. Sections were incubated with primary antibody diluted in blocking solution overnight at 4°C. The primary antibodies used were goat anti-doublecortin (DCX) (Santa Cruz, SC-8066; 1:200; Antibody Registry ID AB_2088494), mouse anti-NeuN (Millipore, MAB377; 1:500; Antibody Registry ID AB_10048713), rat anti-CD11b (Millipore, CBL1313; 1:500; Antibody Registry ID AB_92930), rat anti-CD16/32 (BD Pharmingen, 553141 Clone 2.4G2; 1:800); rabbit anti-CD206 (Abcam, AB64693; 1:50), rabbit anti-GFAP (Dako, Z0334; 1:500; Antibody Registry ID AB_2314535), rabbit anti-Olig2 (Millipore/Chemicon, AB9610; 1:500), rat anti-Ly-6B.2 (Serotec, MCA771GA; 1:1000; Antibody Registry ID AB_324243). Sections were then washed in PBS and incubated with Alexafluor^488^-conjugated donkey anti-goat, goat anti-mouse, goat anti-rabbit or goat anti-rat IgG (all Molecular Probes; 1:500) diluted in PBS, for 2h at RT. Sections were given a final wash with DAPI (1:5000, Invitrogen D1306) in PBS, slide mounted and coverslipped using Mowiol [49] or Dako fluorescence mounting medium (Dako, Sydney, Australia).

#### Double labelling with EdU

EdU labelling was performed following cell type specific antibody staining detailed above. After secondary antibody labelling, sections were washed in PBS and blocked with 5% bovine serum albumin (Sigma) and 0.5% Triton X-100 (Sigma) in PBS for 30min. Sections were washed in PBS and incubated in a reaction cocktail prepared from the Click-iT® EdU Alexa Fluor® 555 Imaging Kit (Molecular probes (Life technologies), Melbourne, Australia) for 45min at 4°C. Components of the Click-iT® reaction cocktail were prepared as per manufacturer’s instructions however a reduced total volume of 300μl per tissue well was used. Also, the Alexa Fluor® 555 azide dilution was increased by dissolving the stock in 140μl of DMSO instead of 70μl and using 1.25μl of working stock per ml of reaction cocktail instead of 2.5μl. Additionally, component D (1x Click-iT® reaction buffer) was replaced with PBS. Sections were given a final wash with DAPI in PBS and slide mounted as above.

#### Hematoxylin and Eosin (H&E) staining for analysis of lesion areas

Slide mounted frozen sections were thawed and stained with H&E. Slides were air dried for 30sec, fixed in 10% neutral buffered formalin for 1min and placed in haematoxylin for 30sec. Slides were then washed in tap water, placed in Scott’s tap water for 30sec and again washed in tap water before placing in 1% eosin for 30sec. Slides were given a quick wash in water and then dehydrated in 100% ethanol for 1min and moved to fresh 100% ethanol twice for 30sec each. Lastly, slides were cleared in xylene for 3min and moved to fresh xylene twice for 2min each before coverslipping with D.P.X. mounting medium (Sigma).

### Imaging, cell counting and analysis of lesion areas

All standard fluorescence and bright field imaging was performed using an Olympus 1X81 inverted microscope with a mercury burner and white light source. Cell counts were performed using ImageJ (NIH, Maryland, USA) with the aid of the cell counter plugin and the hyper-stack channel tool for determining co-localisation of different cell markers and EdU.

For sections at the 35d timepoint, for each mouse three frames at x20 magnification were captured of the ipsilateral cortex around the lesion edge and also three frames at equivalent regions of the contralateral cortex. Counts from the three frames were totalled for each side of the cortex and converted to mm^2^. Total counts from 6 serial sections spaced 300μm apart were averaged for each animal, or counts from each serial section were averaged per group to assess numbers across the injury site from bregma position −600μm to 900μm. For a group the results are expressed as cells per mm^2^ ± SEM.

For the 7d timepoint one frame at x4 magnification was captured for each section of the ipsilateral cortex centred at the lesion for analysis of GFAP, CD11b, CD206 and CD16/32 immunohistochemistry. Two overlapping frames at x4 magnification were taken and merged to capture the entire ipsilateral cortex for analysis of EdU immunohistochemistry, using the same exposure times and imaging settings for each section. Images were analysed using the threshold function on Image J. A threshold was set for each image such that it covered the entire stained area of the ipsilateral cortex. The area covered by the threshold in the ipsilateral cortex was then measured. Areas of 6 matching serial sections were averaged to obtain an average lesion area over the injury site from bregma −600μm to 900μm. For a group the results are expressed as ipsilateral cortical area covered by threshold (mm^2^). Two frames at x20 magnification, one either side of the lesion were taken for analysis of Olig2, Dcx and Neutrophil immunohistochemistry. Images were analysed by totalling the counts from each frame per section and converting to mm^2^. Total counts from 6 serial sections spaced 300μm apart were averaged for each animal or counts from each serial section were averaged per group. For a group the results are expressed as the cells per mm^2^ ± SEM of ipsilateral cortex.

For determining lesion area of all TBI tissue from H&E, bright field images at 4x magnification were obtained of the lesion site. ImageJ was used to calculate the area by tracing damaged or abnormal looking tissue in the ipsilateral cortex. Areas of 6 matching serial sections were averaged for a group to obtain an average lesion area every 300μm over the injury site from bregma position −600μm to 900μm.

### Statistical analysis

GraphPad Prism 5 was used for all statistical analyses and graph generation. Data was analysed using the un-paired t-test for comparison of two groups or 2-way analysis of variance (ANOVA) for comparison of genotype and injury effects. Individual comparisons were performed using Bonferroni post-hoc testing, with n = 4–8 mice/group. For all experiments results were considered significant when *p*< 0.05.

## Results

SOCS2Tg and WT mice were subjected to TBI, administered a daily dose of EdU for 7d and immunohistological and behavioral analyses were performed to assess effects of SOCS2 overexpression on outcome. Initial studies were performed using a mild injury paradigm to examine neurogenesis at 35d post-injury. However, as described below, the mild injury model resulted in little/no production of newborn neurons nor did it produce measurable behavioral effects, although effects on the macrophage/microglial response and astrocytic gliosis in peri-lesional cortex were observed. Subsequent studies used a moderately severe paradigm, which resulted in a modest forepaw motor deficit and detectable injury induced neurogenesis at 35d post-injury, as well as an enhanced inflammatory response to injury. Due to the lack of newborn neurons in the mild injury paradigm, the moderately severe paradigm also included cohorts that were administered EPO as a potential neuroprotective agent. EPO has been show to promote neurogenesis following neural damage [24] and was included in the study to determine whether an additional stimulus was required to enhance newborn neuron survival. Finally, a cohort of mice was given moderately severe injury and tissues were analyzed at 7d rather than 35d post-injury, to further explore effects of SOCS2 overexpression, including potential earlier effects on neurogenesis and neuroinflammation to try to uncover a mechanism by which SOCS2 overexpression modulates to response to TBI.

### SOCS2Tg animals displayed an improved motor function by 7d post moderately severe TBI

**Fig 1:**
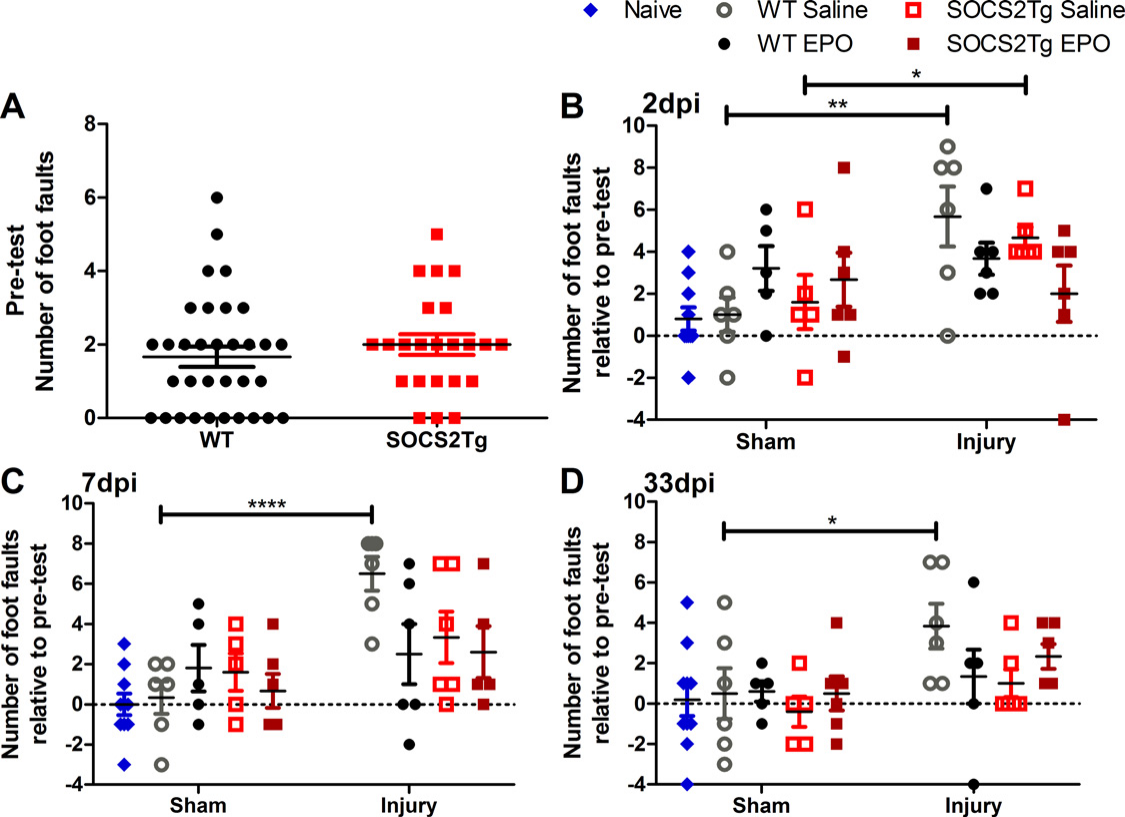
SOCS2 overexpression or EPO improve functional outcome following moderately severe TBI. The number of foot faults made across a 100cm ladder by the right forepaw of SOCS2Tg and WT mice was examined using the horizontal ladder 14 days pre-injury and 2, 7 & 33 days post injury (dpi). All mice made right forepaw foot faults prior to injury but no genotype differences were present (A). At 2dpi only injured WT and SOCS2Tg saline treated mice showed a significant increase in foot faults compared to sham groups (B). At 7d (C) and 33d (D) only injured WT saline treated mice showed significantly greater foot faults compared to sham. Results in B-D show mean foot fault score ± SEM relative to pre-test (Score = Post TBI–Pre TBI); *n* = 23–33 mice/group (pre-injury) and *n* = 5–10 mice/group (2, 7 & 33dpi); *P<0.05, **P<0.01, ****P<0.0001 (ANOVA with Bonferroni post hoc test; B-D).

The mild TBI model did not produce motor deficits, however the moderately severe model produced mild functional impairment of the right forepaw but did not affect anxiety-like behavior (elevated plus maze) or spatial memory (Y maze) (data not shown). To determine whether SOCS2 overexpression modified functional outcome and whether this could be further modified by EPO administration, SOCS2Tg and WT animals underwent motor function testing using the horizontal ladder test 2 weeks prior to injury and at 3, 7 and 33d post moderately severe-TBI. A naïve WT animal group was also tested together with the sham and injured groups at these time points. Testing prior to injury showed that both SOCS2Tg and WT animals made foot faults in the uninjured state and this was not different between genotypes (Fig 1A). While the average number of foot faults pre-injury for both groups was 2, there was a large variation between individual animals ranging from 0 to 6 foot faults within each group. Given this large variation in pre-injury foot fault number, foot fault data was analyzed relative to pre-test following TBI. There was a significant effect of injury at 2d (*F*_(1,38)_ = 5.8; *P* = 0.0209), 7d (*F*_(1,37)_ = 11.18; *P* = 0.0019) and 33d (*F*_(1,38)_ = 7.2; *P* = 0.0107) post-injury. WT saline control mice showed continuing functional deficit until at least 33d post-injury, while SOCS2Tg saline treated mice showed functional deficit at 2d but this returned to control levels by 7d (Fig 1B–1D). Injured SOCS2Tg and WT EPO treated mice showed no significant functional deficits compared to sham EPO treated mice at any time point post-injury and EPO did not further improve functional outcome in SOCS2Tg mice. Therefore, SOCS2 improved functional outcomes and appeared to do so independently of EPO administration.

### SOCS2Tg mice had a smaller lesion area compared to WT at 7d but not 35d post injury

**Fig 2:**
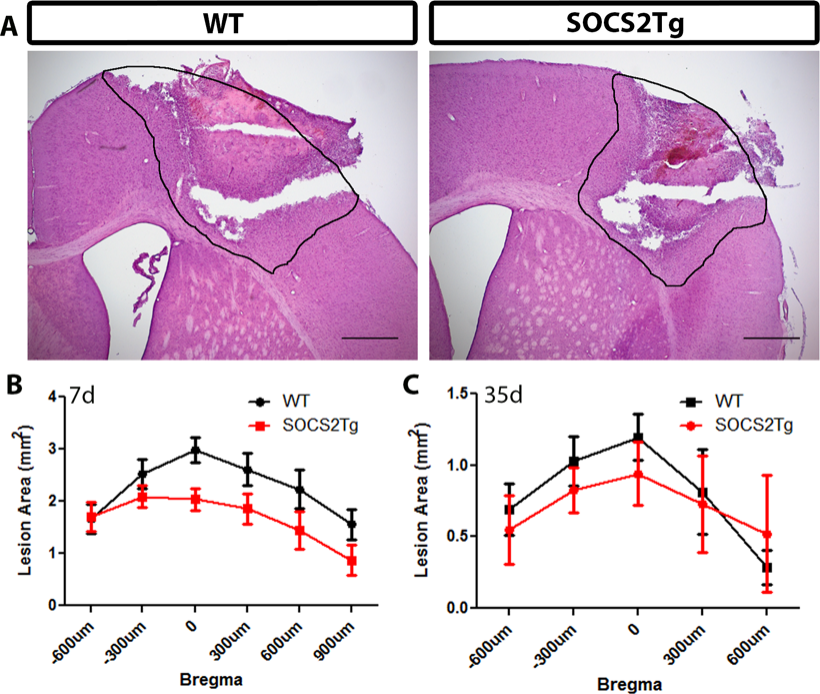
SOCS2Tg mice had a smaller lesion size than WT mice 7d post moderately-severe TBI. The lesion area of injured SOCS2Tg and WT mice was assessed by haematoxylin and eosin staining of coronal tissue sections. Representative images, indicating the traced lesion area, from injured WT and SOCS2Tg mice are shown (A). At 7d SOCS2Tg mice had a smaller lesion, with a significant effect of genotype on lesion area between genotypes (*F*_(1,73)_ = 12; *P* = 0.0008; Two-way ANOVA) but no significant differences were found in post hoc analysis (B). By 35d the lesion area was smaller than at 7d and there was no significant difference between genotypes. Results show mean area ± SEM; *n* = 5–8 mice/group (B, C). Scale bars in A = 500μm.

The cortical lesion area of SOCS2Tg and WT mice was measured at 7d and 35d post moderately severe injury after hematoxylin and eosin (H and E) staining of coronal tissue sections. At 7d post injury measurements confirmed a peak lesion area at zero bregma and revealed a statistically significant effect of genotype (*F*_(1,73)_ = 12; *P* = 0.0008): SOCS2Tg mice showed a smaller lesion area compared to WT mice. However, no significant differences were found at specific bregma points between genotypes in post hoc analysis (Fig 2A and 2B). At 35d post-injury there was no significant effect of genotype (Fig 2C) or EPO treatment (S1 Fig) on lesion area throughout the lesion site, although there was a trend (*P* = 0.068) to decreased lesion area in SOCS2Tg saline treated mice compared to WT.

### SOCS2Tg mice had greater injury-induced cell proliferation in the cortex than WT mice after mild but not moderately severe TBI

**Fig 3:**
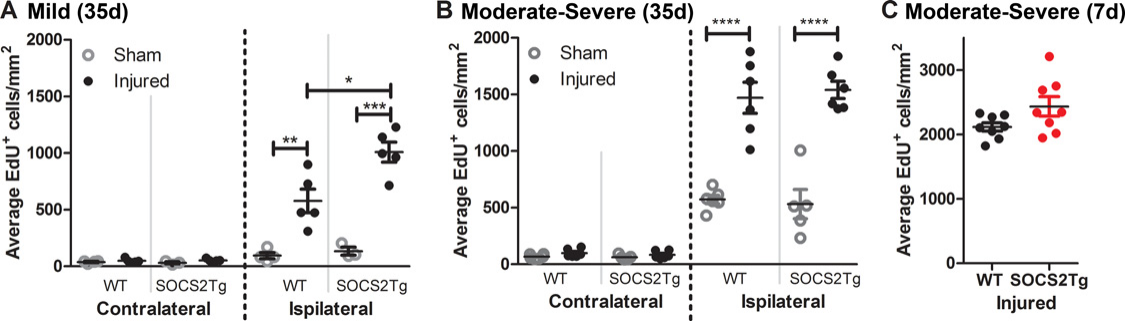
SOCS2 overexpression increased numbers of proliferative cells in injured cortex compared to WT after mild but not moderately severe TBI. The perilesional cortex of SOCS2Tg and WT animals was analysed 35d post TBI or sham surgery for presence of EdU^+^ cells. In injured mice there was a significant increase in EdU^+^ cells in the ipsilateral cortex and after mild TBI (A) this increase was 2 fold greater in SOCS2Tg mice compared to WT. While there was an injury induced increase in proliferative cells after moderately severe TBI (B), there was no effect of SOCS2 overexpression There was also no significant effect of SOCS2 overexpression on proliferative cell numbers at 7d post moderately severe TBI (C). Results show mean ± SEM; *n* = 5–8 mice/group **P<0.01, ***P<0.001, **** *P*<0.0001 (ANOVA with Bonferroni post hoc test).

Assessment of injury-induced cell proliferation in the cortex was analyzed by EdU^+^ cell counts. A statistically significant effect of injury was found for both mild (*F*_(3,26)_ = 83.17; *P* = <0.0001) and moderately severe TBI (*F*_(7,76)_ = 175; *P*<0.0001) at 35 days post-TBI, with SOCS2Tg and WT injured mice having greater numbers of EdU^+^ cells in the ipsilateral cortex compared to sham mice (Fig 3A and 3B). Further, a statistically significant effect of genotype (*F*_(1,26)_ = 7.34; *P* = 0.0118) and a significant interaction between injury and genotype (*F*_(3,26)_ = 6.92; *P* = 0.0014) was observed for mild TBI. Post hoc analysis revealed that SOCS2Tg mildly injured mice had greater number of EdU^+^ cells in the ipsilateral cortex compared to WT injured mice but no effect of genotype was observed after moderately severe injury. Further, EPO had no effect on cell proliferation (S2 Fig) and no differences between groups were observed in the contralateral cortex at either injury severity. To determine whether there was an earlier effect on cell proliferation in the moderately severe injury group, analysis of EdU^+^ cells in the injured cortex was also performed at 7d post-injury. No significant effect of genotype was observed, although there was a trend to increased numbers in the SOCS2Tg brains (*P* = 0.07) (Fig 3C).

The analyses below assess the contributions of different neural cell types to the EdU labelled population to determine which cells were responsible for the genotype and TBI-induced proliferative response.

### SOCS2 overexpression had no effect on cortical neurogenesis after TBI

**Fig 4:**
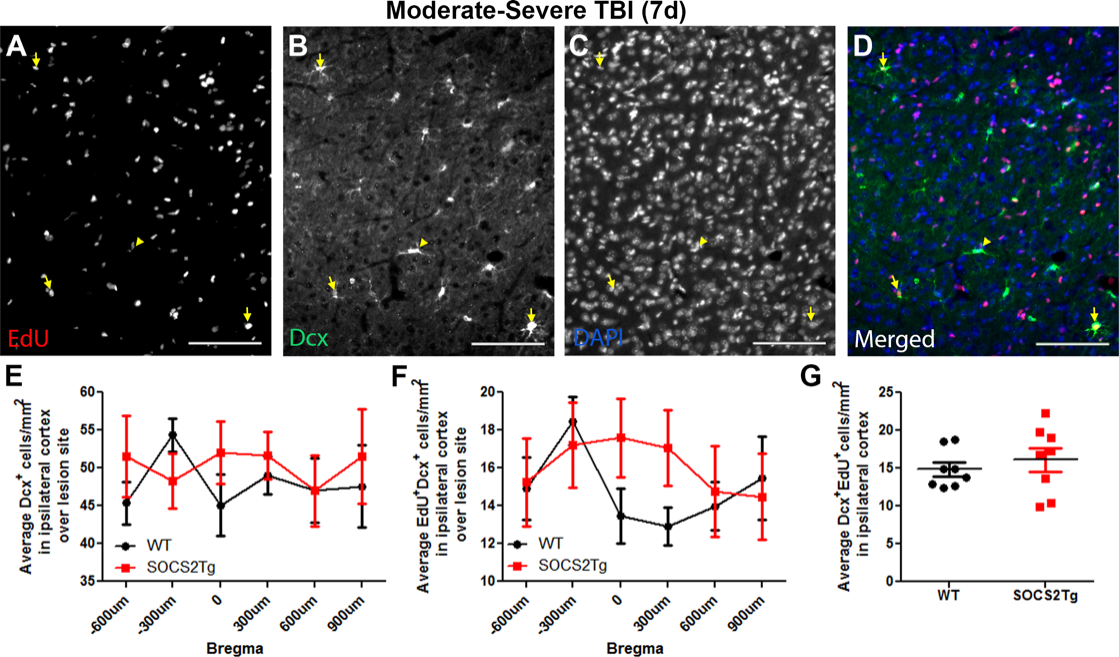
SOCS2 overexpression had no effect on neuroblast generation after TBI. The perilesional cortex of SOCS2Tg and WT mice was analysed after moderately-severe TBI at 7d for EdU^+^Dcx^+^ newborn neuroblasts. EdU^+^Dcx^+^ cells were present in both WT and SOCS2Tg perilesional cortex and a representative image of SOCS2Tg cortex is shown (A-D). Panel D is the merged image of panels A-C. Scale bars = 100 μm. Examples of co-labelled cells are indicated by yellow arrows and a Dcx+/EdU- cell by the yellow arrowhead. No genotype differences in Dcx+ cell number (E), EdU^+^Dcx^+^ cell number across Bregma position (F) or average numbers of EdU^+^Dcx^+^ cells (G) were seen. Results show mean ± SEM; n = 7–8 mice/group (E-G).

**Fig 5:**
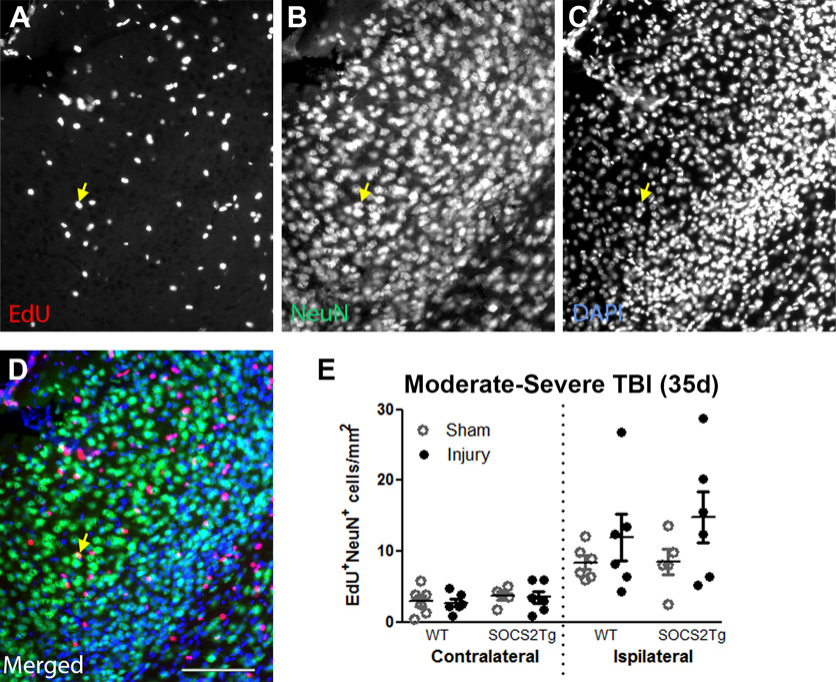
SOCS2 overexpression had no effect on newborn neuron survival after TBI. The perilesional cortex of saline or Epo-treated SOCS2Tg and WT mice was analysed after moderately-severe TBI at 35d for EdU^+^NeuN^+^ newborn neurons. EdU^+^NeuN^+^ cells were present in both WT and SOCS2Tg perilesional cortex and a representative image of SOCS2Tg saline control cortex is shown (A-D). Panel D is the merged image of panels A-C. Scale bars = 100 μm. An example of a co-labelled cell is indicated by the arrow. No genotype differences in numbers of EdU^+^NeuN^+^ cells (E) were seen. Results show mean ± SEM; n = 7–8 mice/group (E).

To determine whether SOCS2 overexpression had an effect on generation of neuroblasts or survival of mature newborn neurons, brains were immunostained at 7d for the immature neuron marker Doublecortin (Dcx) (Fig 4) or at 35d for the mature neuron marker NeuN (Fig 5) and EdU.

Dcx^+^ cells were present in approximately equal numbers throughout the area of perilesional cortex 7d after injury and there were no overall differences between SOCS2Tg and WT mice, except for a modest increase in the number of EdU^+^/Dcx^+^ cells in the middle of the SOCS2Tg lesion site compared to WT (0 and 300μm Bregma points combined; *p*<0.01) (Fig 4E–4G). Newly generated EdU^+^Dcx^+^ cells made up 25–35% of the Dcx^+^ cell population in the perilesional cortex of both genotypes, confirming that many neuroblasts are generated after injury and recruited to the lesion site.

In the moderately severely injured mice a small number of EdU^+^NeuN^+^ cells were identified in both the ipsilateral and contralateral cortex of all groups (Fig 5E). However, a large variation in newborn neuron number was present within the ipsilateral cortex of each group and no significant differences were observed between genotype and/or EPO treatment (S3 Fig). A significant effect of injury was present (F_(7,75)_ = 12; P = <0.0001), however post-hoc analyses did not show differences between sham and injured group ipsilateral cortices.

### SOCS2 overexpression enhanced astrocyte proliferation compared to WT after mild TBI at 35d and after moderately severe TBI at 7d

**Fig 6:**
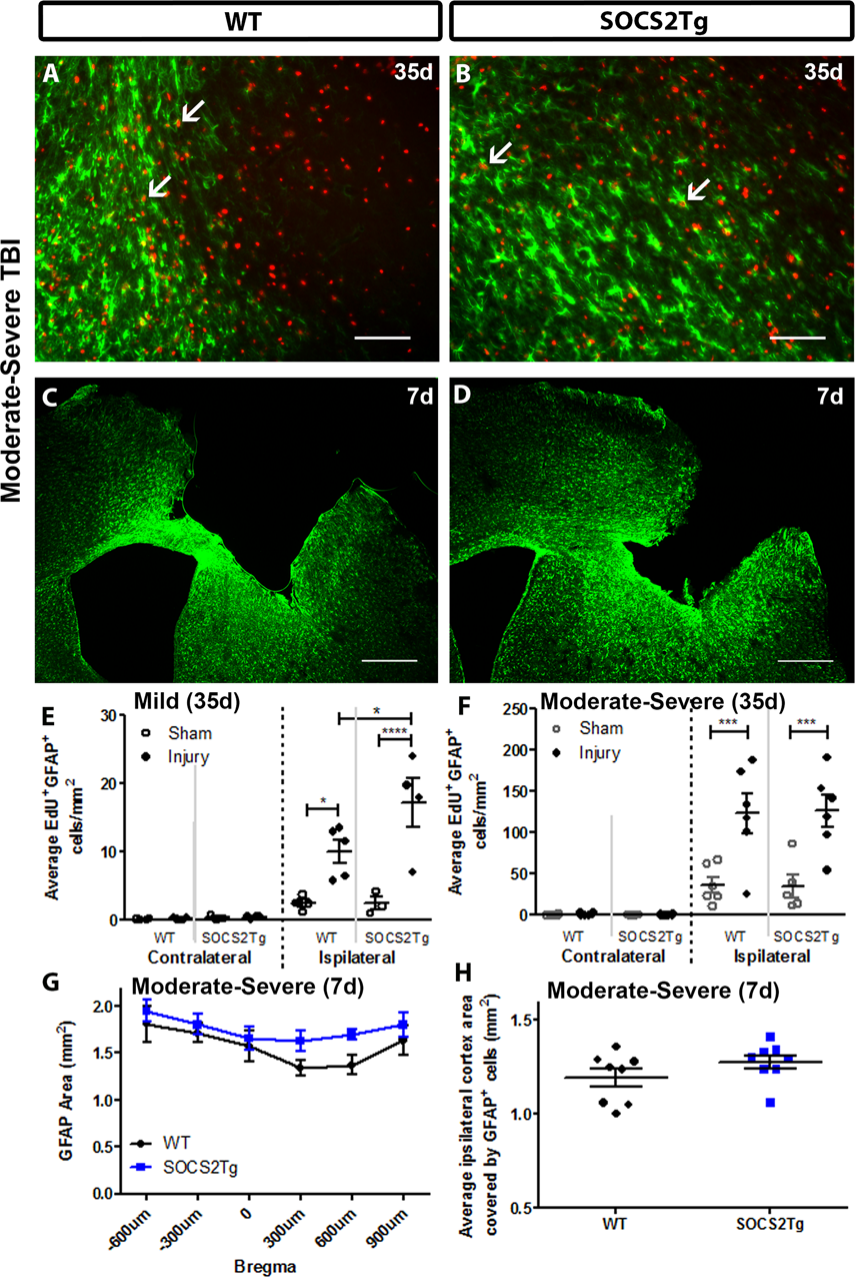
SOCS2 overexpression increased numbers of proliferative astrocytes in injured cortex compared to WT after mild but not moderately severe TBI. The perilesional cortex of SOCS2Tg and WT mice was analysed at 7d and 35d post TBI or after sham surgery for density of EdU^+^GFAP^+^ cells. Representative images of WT (A, C) and SOCS2Tg (B, D) cortex are shown. Scale bar A,B = 100 μm; C,D = 500 μm. Examples of co-labelled cells in injured cortex after moderately severe TBI are indicated by arrows in panels A and B and low power images used for thresholding cortical area covered by GFAP at 7d post-TBI are in panels C and D. In the ipsilateral cortex of SOCS2Tg and WT injured animals there was an overall injury-induced increase in EdU^+^GFAP^+^ cell density at 35d post mild (E) and moderately severe TBI (F), which was further enhanced in SOCS2Tg mice after mild TBI (E). At 7d after moderately severe TBI, the area covered by GFAP expression was measured by thresholding using ImageJ. There was an overall increase in GFAP area in SOCS2Tg mice when measured across Bregma levels (G) which was not significant when sections from each animal were combined and averaged (H). Results show mean ± SEM; n = 5–8 mice/group (E-H); *P<0.05, **** P<0.0001 (ANOVA with Bonferroni post hoc test).

The astroglial response was assessed by staining for EdU and the astrocyte marker GFAP (Fig 6). Quantification of EdU^+^GFAP^+^ cells showed that injury significantly increased the numbers of proliferative astrocytes in the ipsilateral cortex at 35d following mild (*F*_(3,24)_ = 38.02; *P* = <0.0001) and moderately severe TBI (*F*_(7,76)_ = 33; *P* = <0.0001) compared to sham animals. Following mild injury, there was a further increase in the SOCS2Tg ipsilateral cortex compared to WT, but not after moderately severe injury (Fig 6E and 6F), with or without EPO treatment (S4 Fig). No or very few EdU^+^GFAP^+^ cells were observed in the contralateral cortex of sham and injured mice (data not shown). At 7d after moderately severe TBI the GFAP^+^ cell response was analyzed by measuring the thresholded area of the immunostained perilesional cortex of SOCS2Tg and WT mice using Image J. This method of analysis was chosen due to the high density of astrocyte staining at this early and more severe post-TBI time point. An effect of genotype (F_(1,81)_ = 7.2; P = 0.0089) and bregma (F_(5,81)_ = 3; P = 0.0143) was present for the GFAP^+^ cell response at 7d post injury but no specific differences were identified by post hoc analysis (Fig 6G and 6H). The effect of genotype most likely reflects the trend seen for a greater GFAP^+^ area from bregma 300 to 900 μm in SOCS2Tg mice.

### SOCS2 overexpression did not affect oligodendrogliogenesis after mild or moderately severe TBI

**Fig 7:**
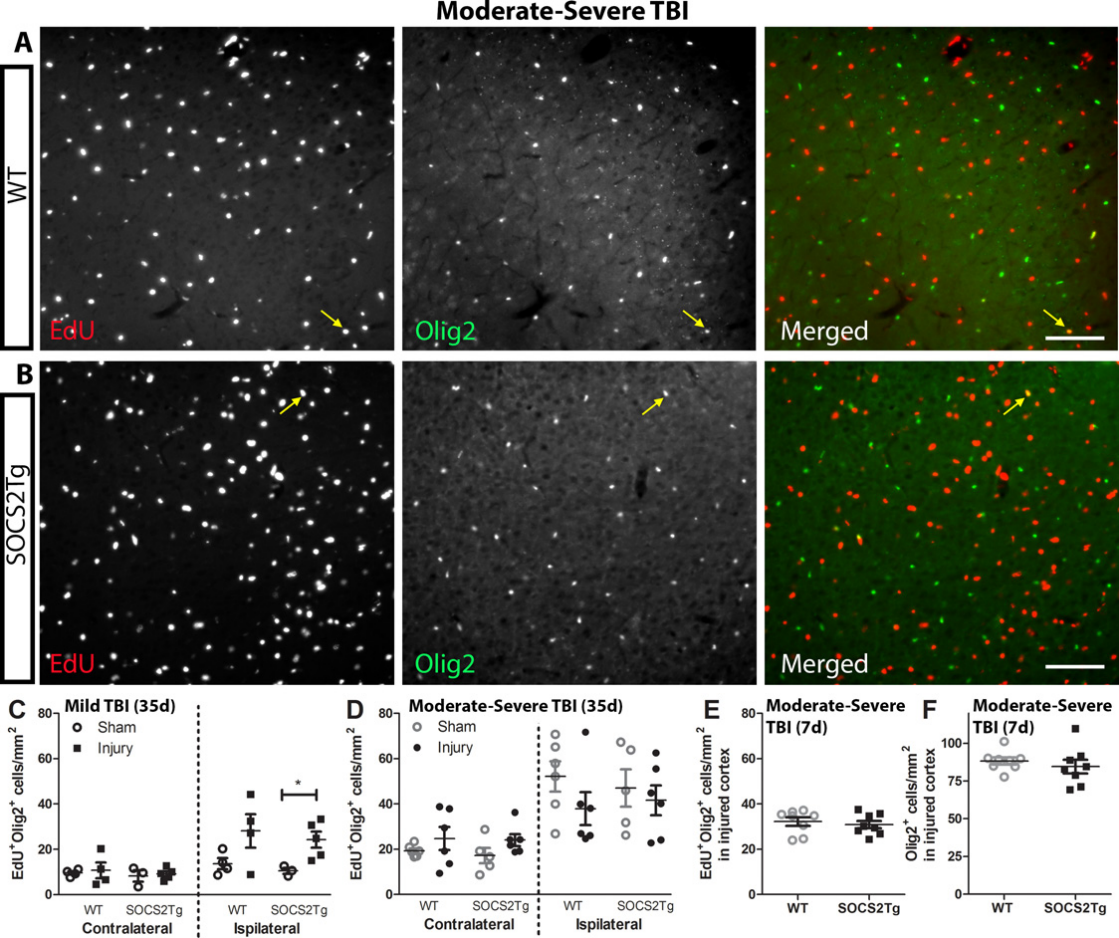
SOCS2 overexpression had no effect on generation of oligodendrocytes. The perilesional cortex of SOCS2Tg and WT mice was analysed at 7d and 35d post TBI or sham surgery for density of EdU^+^Olig2^+^ cells. Representative images of WT (A) and SOCS2Tg (B) cortex are shown after moderately severe injury are shown. Scale bar A,B = 100 μm. The merged panels are the EdU and Olig2 panels combined. Examples of co-labelled cells in injured cortex are indicated by arrows in panels A and B. In the ipsilateral cortex of SOCS2Tg and WT injured animals there was an overall injury-induced increase in EdU^+^Olig2^+^ cell density at 35d post mild TBI (C) which was further enhanced after moderately severe TBI (D) but with no effect of genotype. Further, there was no effect of genotype on EdU^+^Olig2^+^ (E) or Olig2^+^ (F) cell density in injured cortex at 7d post moderately severe TBI. Results show mean ± SEM; n = 3–8 mice/group.

The oligodendroglial response was assessed by staining for EdU and Olig2 (oligodendrocyte lineage marker; EdU^+^Olig2^+^ cells represent proliferative oligodendrocyte precursor cells or their more mature progeny) (Fig 7). Quantification of EdU^+^Olig2^+^ cells revealed a statistically significant effect of injury following mild (*F*_(3,24)_ = 11.06; *P* = <0.0001) and moderately severe TBI (F_(7,76)_ = 14; P = <0.0001) but there was no effect of genotype at 35d post-injury (Fig 7A–7D). Few EdU^+^Olig2^+^ cells were observed in the contralateral cortex of sham and injured animals. Further, no significant differences were present between SOCS2Tg and WT mice in the number of EdU^+^Olig2^+^ or Olig2^+^ cells in the perilesional cortex at 7d post injury (Fig 7E and 7F). For both SOCS2Tg and WT mice, EdU^+^Olig2^+^ cells made up 30–40% of the Olig2^+^ cell population in the perilesional cortex (data not shown).

### SOCS2Tg mice displayed enhanced microglial/macrophage proliferation after a mild but not moderately severe TBI

**Fig 8:**
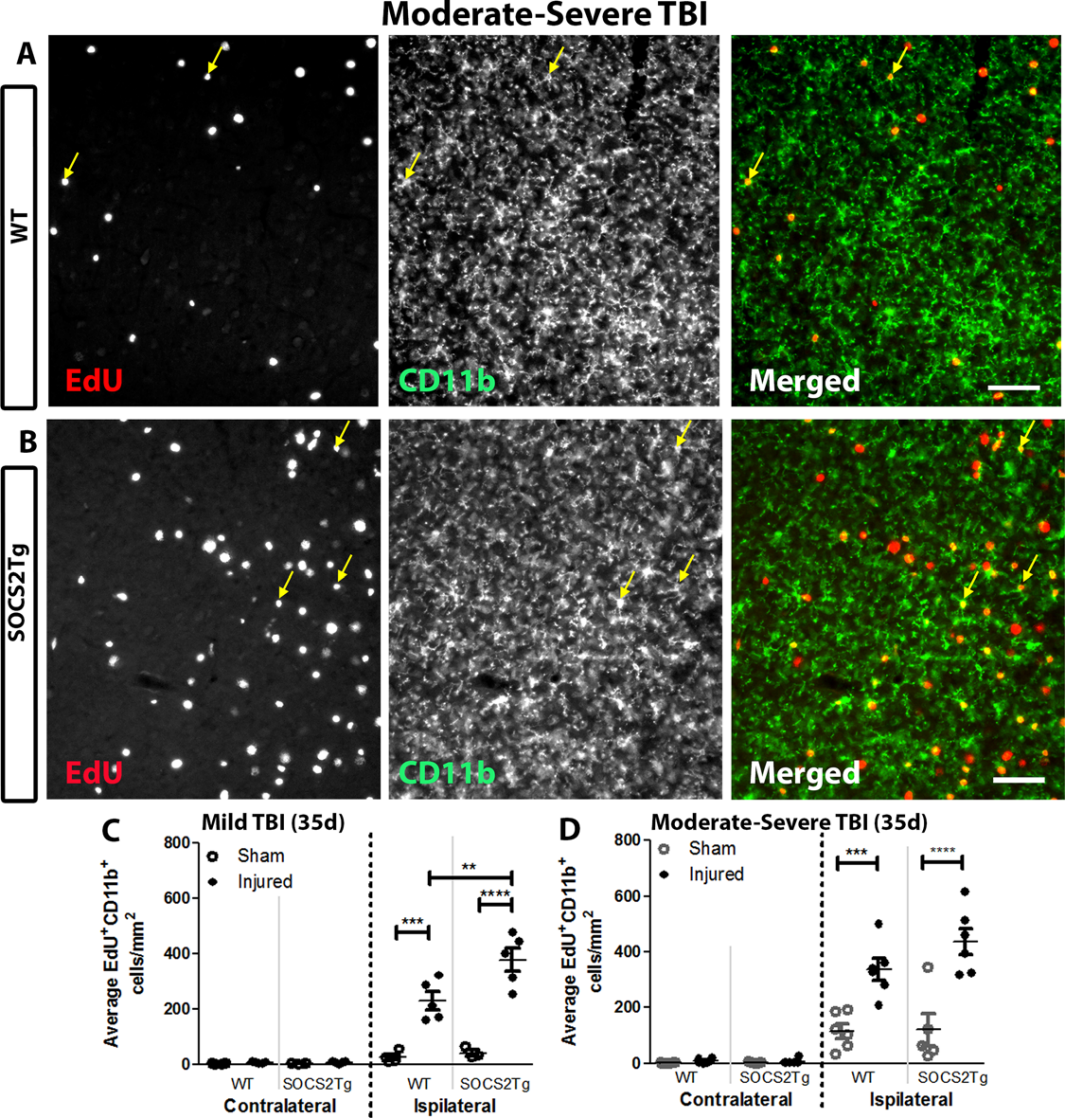
SOCS2 overexpression increased numbers of proliferative macrophages/microglia at 35d post-injury in injured cortex compared to WT after mild but not moderately severe TBI. The perilesional cortex of SOCS2Tg and WT saline and Epo-treated mice was analysed at 35d post TBI or sham surgery for density of EdU^+^CD11b^+^ cells. Representative images of WT (A) and SOCS2Tg (B) cortex after mild TBI are shown. Scale bar A,B = 40 μm. Examples of co-labelled cells in injured cortex are indicated by arrows in panels A and B. There was an injury-induced increase in EdU^+^CD11b^+^ cells in the ipsilateral compared to contralateral cortex (C,D) which was enhanced in SOCS2Tg mice compared to WT after mild TBI (C). Results show mean ± SEM; *n* = 5–8 mice/group (C,D). **P<0.01, ***P<0.001, **** P<0.0001 (ANOVA with Bonferroni post hoc test).

The neuroinflammatory response was assessed by staining for EdU and the activated macrophage/microglial marker CD11b (Fig 8A and 8B). Quantification of EdU^+^CD11b^+^ cells at 35d post-injury revealed a statistically significant effect of injury after mild (*F*_(3,26)_ = 91.86; *P* = <0.0001) and moderately severe (*F*_(7,76)_ = 51.80; *P* = <0.0001) injury, with SOCS2Tg and WT injured animals having a greater number of EdU^+^CD11b^+^ cells in the ipsilateral cortex compared to sham. No or few EdU^+^CD11b^+^ cells were observed in the contralateral cortex of all animals. A significant effect of genotype (*F*_(1,26)_ = 6.390; *P* = 0.0179) and a significant interaction between injury and genotype (*F*_(3,26)_ = 5.498; *P* = 0.0046) was also observed for the EdU^+^CD11b^+^ cell response after mild injury: SOCS2Tg injured mice had a greater number of EdU^+^CD11b^+^ cells in the ipsilateral cortex compared to WT animals (Fig 8C). Therefore, the larger EdU^+^ cell population in mildly injured SOCS2Tg injured mice was most likely a reflection of increased macrophage/ microglial proliferation compared to WT injured mice. After moderately severe injury SOCS2 over-expression (Fig 8D) and/or EPO (S5 Fig) had no significant effect on numbers of EdU^+^CD11b^+^ cells at 35d post-injury.

### Increased M2-like but not M1-like macrophages/ microglia in the perilesional cortex in SOCS2Tg mice at 7d post injury

**Fig 9:**
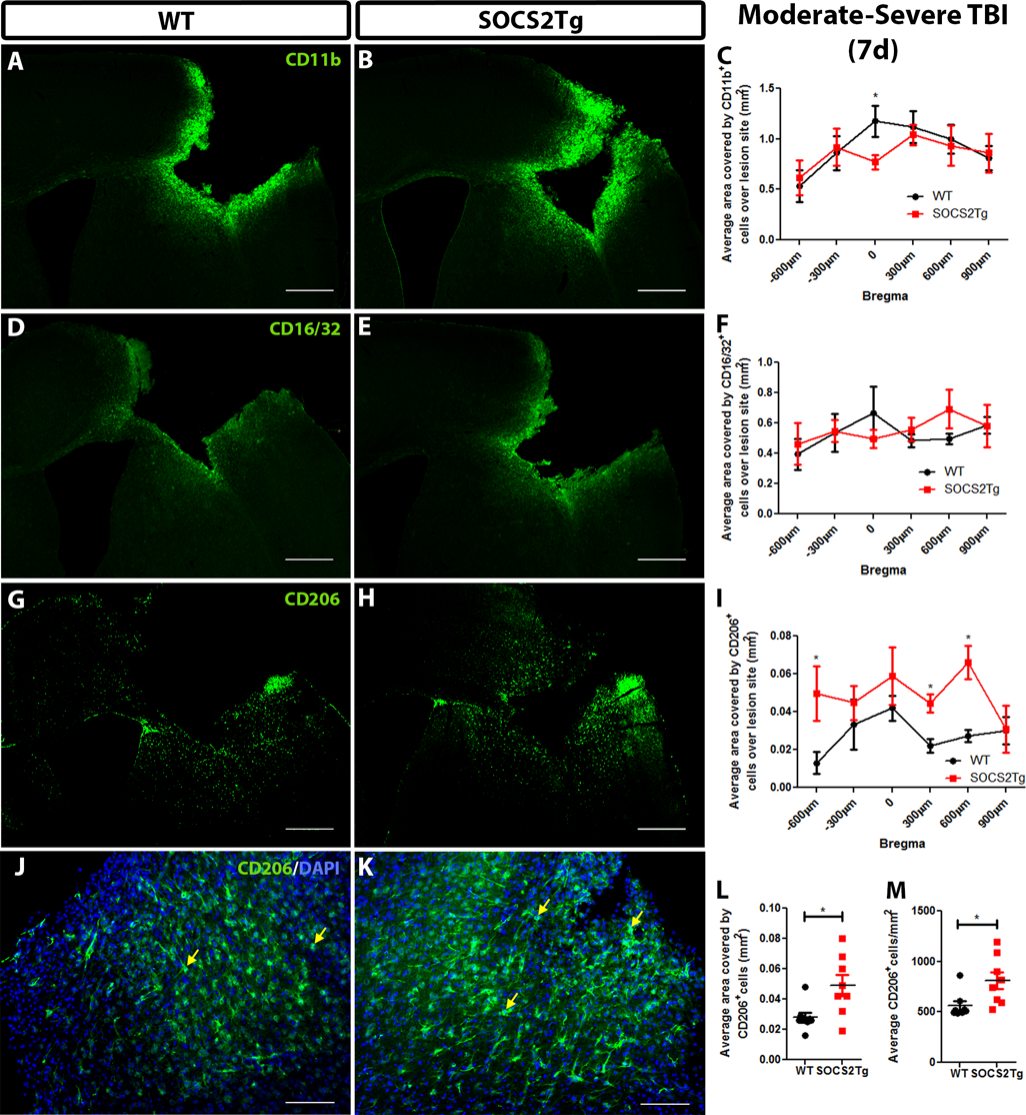
SOCS2Tg mice had greater numbers of CD206^+^ cells 7d post moderately-severe TBI. The macrophage/microglial cell response in SOCS2Tg and WT mice was assessed 7d post moderately-severe TBI. Representative images of ipsilateral coronal tissue sections through the injury site, stained with CD11b (A, B), CD16/32 (D,E) and CD206 (G,H,J,K) are shown for WT (A,D,G,J) and SOCS2Tg (B,E,H,K) mice; scale bar = 500μm (A,B,D,E,G,H) and 100 μm (J,K). Examples of CD206^+^cells are indicated by arrows. No effect of genotype or bregma position was seen for CD11b (C) or CD16/32 (F) expression. SOCS2Tg mice showed a significantly greater area covered by CD206^+^ cells compared to WT mice both across bregma position (I) and averaged per brain (L) as well as increased average CD206^+^ cell density (M). Results show mean ± SEM; *n* = 7–8 mice/group; *P<0.05 (ANOVA with Bonferroni post hoc; C,F,I,L). (C,I) or unpaired t-test (L,M).

Mildly injured SOCS2Tg mice displayed an altered inflammatory cell response 35d post TBI and moderately injured SOCS2Tg displayed improved motor function outcome at 7d post. This suggested that the effect of SOCS2 was early and that an inflammatory mechanism was a possible contributor. To examine if there was alteration in inflammatory cell responses of SOCS2Tg mice at 7d post moderately-severe TBI, tissue was immunostained for Ly-6B.2 (Neutrophils), CD11b (general macrophage/microglia marker), CD16/32 (pro-inflammatory M1 macrophage/microglia marker) or CD206 (anti-inflammatory M2 macrophage/microglia marker) (Fig 9).

A small number of infiltrating neutrophils were seen in the perilesional cortex of both SOCS2Tg and WT mice. Neutrophils were scattered in similar numbers throughout the lesion site and no genotype differences were observed (data not shown). The CD11b^+^, CD16/36^+^ and CD206^+^ cell responses were analyzed by measuring the thresholded area of the stained perilesional cortex of SOCS2Tg and WT mice using Image J. This method of analysis was chosen due to the high density of macrophage/microglial staining at this early timepoint. The area covered by CD11b^+^ cells was not found to be significantly different between genotypes (Fig 9A–9C). There was an effect of bregma position (*F*_(5,84)_ = 2.5; *P* = 0.0365) with higher levels of CD11b^+^ staining near the injury core at zero bregma compared to the lesion edge. CD16/32 staining also showed no significant effect of genotype and no effect of bregma given the similar levels of staining throughout the lesion site (Fig 9D–9F). CD206 staining was lower density than CD16/32 and an effect of genotype was present; post hoc analysis revealed a significantly greater number of and area covered by CD206^+^ positive cells compared to WT (*F*_(1,84)_ = 15; *P* = 0.0002)(Fig 9G–9M). No specific staining was observed in the contralateral cortex (data not shown).

## Discussion

SOCS2Tg and WT animals were subjected to a mild or moderately severe TBI and the neuronal, neuroinflammatory and glial cell responses were analyzed at 7 and 35d post injury. It was hypothesized that SOCS2Tg animals would show enhanced survival of newborn neurons in the perilesional cortex and/or alterations in the neuroinflammatory response. While SOCS2 overexpression or EPO administration protected against functional deficits, few or no EdU^+^NeuN^+^ newborn neurons were identified in the cortex of both WT or SOCS2Tg mice and these numbers were not enhanced by administration EPO. EdU^+^ cell numbers were increased by two fold in the injured ipsilateral cortex of SOCS2Tg mice compared to WT mice following mild but not moderately severe TBI. This greater EdU^+^ cell response was primarily attributed to enhanced macrophage/microglial proliferation in injured SOCS2Tg mice, with a similar but less marked effect in the astrocyte response to mild injury. Overall, this study showed a novel role for SOCS2 in neuroinflammatory processes in the injured cortex and that increased levels of SOCS2 do not appear to affect SVZ neural precursor cell-derived adult neurogenesis.

### SOCS2 overexpression or EPO treatment does not enhance newborn neuron survival in the perilesional cortex

Newborn neurons are readily, if transiently, seen in the injured striatum of rats after stroke induced by middle cerebral artery occlusion (MCAO) [50, 51]. However, there is mixed success reported in the literature regarding presence of newborn neurons at or near the injured cortex after experimental TBI [11–14, 52]. Neural precursor cells (NPCs) are highly sensitive to their environment and the inflammatory microenvironment of the injured cortex is not ideal for NPC survival and their differentiation towards a neuronal fate [53]. Indeed, SVZ derived NPCs have the capacity to generate astrocytes and oligodendrocytes in the presence of specific growth factors *in-vitro* and have been shown to do so preferentially after TBI *in-vivo* [54–56].

TBI-induced neurogenesis was observed in both WT and SOCS2Tg mice, as assessed by presence of Dcx^+^ neuroblasts that migrated from the SVZ towards the injured cortex. There was a modest shift in the distribution of the proliferative neuroblasts at the lesion center, where damage was highest, with decreased numbers of WT cells at that site, but with no overall difference in neuroblast numbers across the lesion site in mice of either genotype. While newborn EdU^+^NeuN^+^ mature neurons were detected in the injured cortex after TBI, their numbers were small and no genotype differences were observed. Therefore, while SOCS2 overexpression can promote survival of SGZ-derived newborn neurons in the hippocampus [22], it does not do so in SVZ-derived neurons in the injured cortex.

Given that the injured cortex is a toxic microenvironment we also administered EPO, due to its reported neuroprotective effects following brain injury [44, 57] and its enhancement of neurogenesis in models of neural damage [46, 58]. While EPO appeared to be protective, given that it improved functional outcome in the ladder test, it had no effect on newborn neuron survival in the current study and did not interact with SOCS2 overexpression to further modify outcome. Indeed, the current study did not reveal a cellular mechanism by which EPO produced functional protection, as it had no differential effect on any of the other cellular parameters examined, including proliferation, oligodendrogenesis, astrogliosis, broad macrophage/microglial responses or lesion volume.

### SOCS2 may have a novel role in the modulation of the macrophage/ microglial cell response after TBI

As expected, TBI induced a robust inflammatory response in SOCS2Tg and WT animals. An injury-induced increase in newly proliferated EdU^+^CD11b^+^ macrophages/ microglia was observed both mild and moderately severe groups at 35d post-injury. However, in the mild injury groups in particular, CD11b^+^ macrophage/microglial proliferation was enhanced in SOCS2Tg injured mice compared to WT. The almost 2 fold difference in EdU^+^CD11b^+^ cells in the SOCS2Tg perilesional cortex compared to WT suggests this may be an important effect of SOCS2 overexpression in the injured cortex. Despite the greater numbers of EdU^+^CD11b^+^ cells in SOCS2Tg mice, the proportion of EdU^+^ cells that were CD11b^+^ was the same between genotypes. In both SOCS2Tg and WT mice approximately 40% of EdU^+^ cells were found to be CD11b^+^ in the injured ipsilateral cortex. Interestingly, while no significant differences were present in the proportion of EdU^+^ cells that expressed CD11b between SOCS2Tg sham and injured mouse ipsilateral cortex, the ipsilateral cortex of WT injured mice showed a significantly greater proportion of EdU^+^CD11b^+^ cells compared to WT sham. This indicates that the severity of TBI normally modulates macrophage/microglial proliferation, with more proliferation associated with more severe injury, but this balance is skewed in SOCS2Tg mice. It is possible that SOCS2Tg animals have either a higher baseline of microglial proliferation, allowing a quicker response to a lower level of stimulation (i.e. mild TBI) or that they have microglia that are better primed to respond to the immune challenge compared to WT animals. If so, then SOCS2Tg cells would reach maximal responsiveness with less stimulation and wildtype cells can catch up to SOCS2Tg levels in a more severe injury (and hence inflammatory) environment.

### SOCS proteins and inflammation

Why the effect of SOCS2 overexpression was more marked in the mild TBI model than the moderately severe model is not clear but may relate to the level of cytokine/inflammatory mediator induction and activation of downstream signaling pathways. Other SOCS family members, namely SOCS1 and SOCS3, have been shown to have important roles in regulating both peripheral and CNS immunity [59]. In terms of CNS macrophage/microglial immune responses, SOCS1 and SOCS3 have also been suggested to play anti-inflammatory roles as they can inhibit interferon or lipopolysaccharide induced MHC class II and CD40 expression on microglia thereby suppressing immune system activity [60, 61]. Encouraging an anti-inflammatory environment after TBI has been associated in some studies with various degrees of improvement in functional outcomes [62, 63].

SOCS2 has been shown to act as an important regulator of anti-inflammatory responses in the periphery and roles for it in directing macrophage polarization towards an anti-inflammatory phenotype have been suggested [37, 64]. Thus, if SOCS2 can exert effects similar to the periphery in the CNS, the enhanced macrophage/microglial proliferation in SOCS2Tg animals may be indicative of an altered inflammatory response after TBI that may potentially be anti-inflammatory in nature. However, an anti-inflammatory effect of SOCS2 overexpression seems counterintuitive given the larger, not smaller number of EdU^+^CD11b^+^ macrophages/microglia at 35d post mild TBI. Studies characterizing the pro (M1) and anti-inflammatory (M2) macrophage/microglial responses after TBI have shown that the initial inflammatory response is dominated by M2-like cells and after 7-14d shifts to M1-like cells [35, 65].

### SOCS2Tg mice had a smaller lesion area and more M2-like macrophages/microglia in the perilesional cortex 7d post moderately-severe TBI

At 7d post moderately-severe TBI, when motor deficit peaked in WT mice, SOCS2Tg mice also had a comparatively smaller lesion area. This was accompanied by an increased number of M2-like (CD206+) anti-inflammatory macrophages/microglia and an equal number of M1-like (CD16/32+) pro-inflammatory macrophages/microglia in SOCS2Tg mice compared to WT. The smaller lesion area at 7d suggests a neuroprotective effect of SOCS2 overexpression after moderately-severe TBI. The mechanism of this potential neuroprotective effect is unclear. However, a greater number of M2-like macrophages/ microglia in the SOCS2Tg perilesional cortex would suggest a mechanism involving the promotion of an anti-inflammatory environment in the acute injury phase, similar to the role of SOCS2 in the peripheral innate immune response [37]. The peak M2 response following TBI has been suggested to occur 3 to 5d following TBI [30, 35, 66]. Therefore, it may be that M2-like macrophages/ microglia were present at even greater numbers in SOCS2Tg mice at earlier time points post-injury. While there is evidence in the literature for SOCS2 regulating anti-inflammatory peripheral immune responses, this is the first study to show a potential effect of SOCS2 on macrophages in the CNS following TBI. In the periphery, SOCS2 deficiency increases neutrophil infiltration into the spleen after infection [37] but no effect of SOCS2 overexpression was seen on neutrophil numbers in the perilesional cortex at 7d post TBI in this study (data not shown).

## Conclusions

This study has described a novel role for SOCS2 in modulation of the macrophage/microglial response following TBI in mice. Overexpression of SOCS2 promoted functional improvement on a ladder test by 7d post-injury, which correlated with a decreased lesion volume and an enhanced anti-inflammatory M2 macrophage/microglial phenotype, however the mechanism by which this occurs remains to be elucidated. This may include secondary effects, such as improved neuronal or mature oligodendrocyte survival due to a more anti-inflammatory environment, which remain to be determined.

Unlike in the hippocampus where SOCS2 overexpression enhances neurogenesis/newborn neuron survival under non-injury conditions [22], SOCS2 does not appear to play a role in regulation of TBI-induced, SVZ-derived adult neurogenesis. However, the neural injury environment is inhibitory for newborn neuron survival. While EPO administration was not successful in overcoming the inhibitory environment, it is possible that with other anti-inflammatory or neuroprotective treatments, a role for SOCS2 in newborn cortical neuron survival may be revealed.

## Supporting Information

S1 Fig EPO had no effect on lesion size and there was no significant difference between genotypes. (TIF)

S2 Fig EPO had no effect on TBI-induced proliferation at 35d post-TBI. (TIF)

S3 Fig EPO had no effect on TBI-induced newborn neuron survival at 35d post-TBI. (TIF)

S4 Fig EPO had no effect on TBI-induced astrocyte proliferation at 35d post-TBI. (TIF)

S5 Fig EPO had no effect on microglial/macrophage activation at 35d post-TBI. (TIF)
