# Supplementary figures and images for: Suppressor of Cytokine Signaling-2 (SOCS2) Regulates the Microglial Response and Improves Functional Outcome after Traumatic Brain Injury in Mice
Source: PLoS One. 2016 Apr 12;11(4):e0153418. doi: 10.1371/journal.pone.0153418 (PMC4829250; doi:10.1371/journal.pone.0153418)

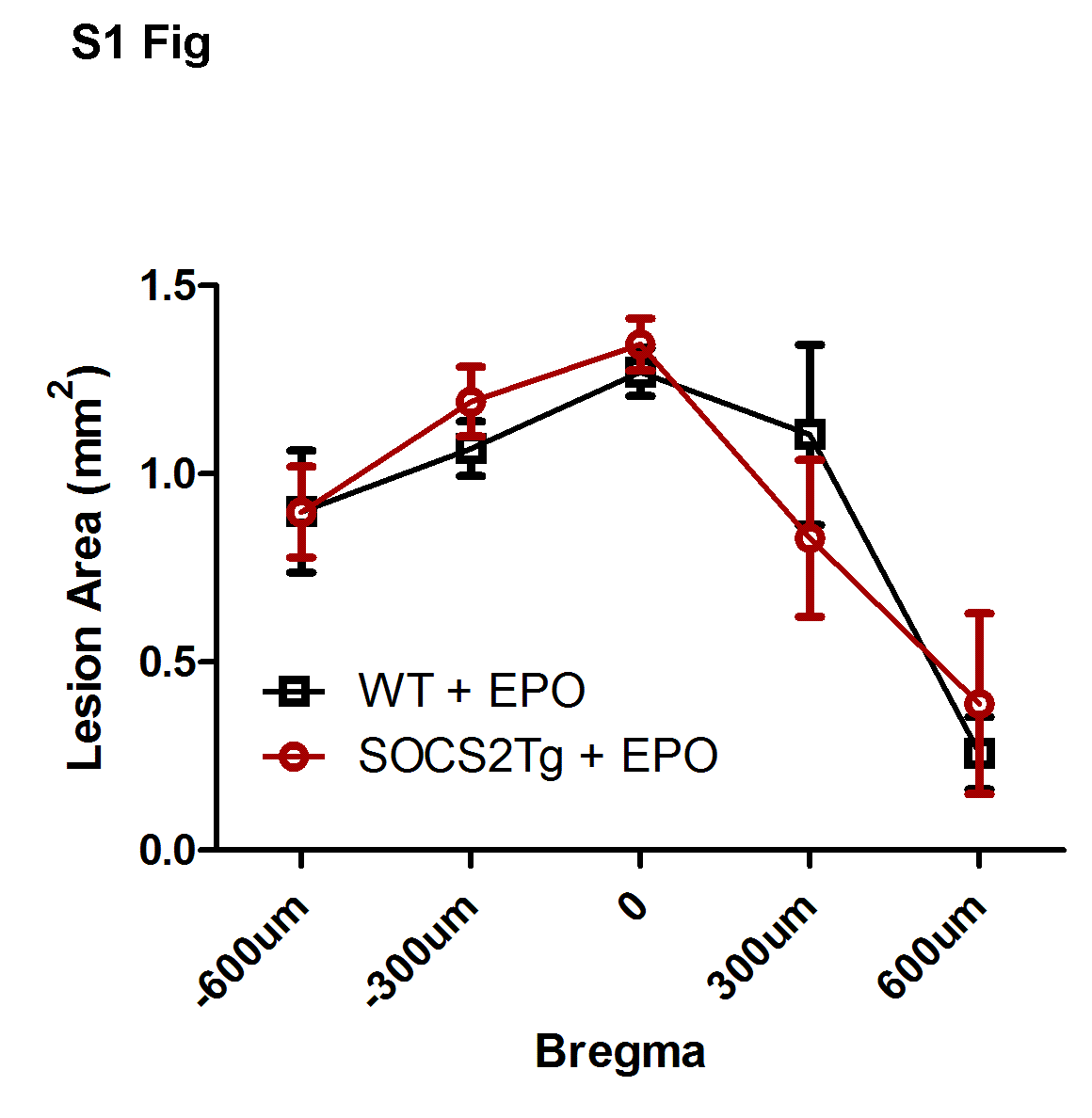

Supplement: S1 Fig — (TIF) [file pone.0153418.s001.tif]
